# Acanmontanoside, a New Phenylethanoid Diglycoside from *Acanthus montanus*

**DOI:** 10.3390/molecules15128967

**Published:** 2010-12-07

**Authors:** Pawadee Noiarsa, Somsak Ruchirawat, Tripetch Kanchanapoom

**Affiliations:** 1 Regional Medical Sciences Center-Khon Kaen, Khon Kaen 40001, Thailand; 2 Chulabhorn Research Institute and Chulabhorn Graduate Institute, Vipavadee-Rangsit Highway, Bangkok 10210, Thailand; 3 Faculty of Pharmaceutical Sciences, Khon Kaen University, Khon Kaen 40002, Thailand

**Keywords:** * Acanthus montanus*, Acanthaceae, acanmontanoside, phenylethanoid glycoside, benzoxazinoid glucoside, aliphatic alcohol glycoside

## Abstract

A new phenylethanoid glycoside acylated with syringic acid, namely acanmontanoside, was isolated from the aerial portions of *Acanthus montanus* (Nees). T. Anderson, along with decaffeoylverbascoside, verbascoside, isoverbascoside, leucosceptoside A, (2*R*)-2-*O*-β-D-glucopyranosyl-2*H*-1,4-benzoxazin-3(4*H*)-one (HBOA-Glc), (2*R*)-2-*O*-β-D-glucopyranosyl-4-hydroxy-2*H*-1,4-benzoxazin-3(4*H*)-one (DIBOA-Glc), (3*R*)-1-octen-3-ol-3-*O*-β-D-xylopyranosyl-(1→6)-*O*-β-D-glucopyranoside and ebracteatoside B. The structure elucidations were based on physical data and spectroscopic analyses including 1D- and 2D-NMR.

## 1. Introduction

As part of our systematic investigation on plants in the genus *Acanthus* of the family Acanthaceae, we reported the constituents of *Acanthus ilicifolius* L., [1,2,3] *A. ebracteatus* Vahl, [4] *A. volubilis* Wall [5]. To further study plants in the same genus, we investigated the chemical constituents of *A. montanus* (Nees) T. Anderson, cultivated at the Botanical Garden of the Faculty of Pharmaceutical Sciences, Khon Kaen University, Thailand. There is no mention on the medicinal uses of this plant in Thai traditional medicine since it is an exotic plant, but its leaves or whole plants are used in African countries for treatment of several ailments such as cough, gastritis, epilepsy, urinary disorders and rheumatic pains. The antimicrobial, anti-inflammatory, analgesic, antipyretic and smooth muscle relaxant properties of the leaf extracts have been reported [6,7,8,9]. Previous phytochemical studies of this plant reported the presence of pentacyclic triterpenoids [10,11]. The present paper describes the isolation and structural determination of polar chemical constituents from the aerial portions of this plant, including a new phenylethanoid glycoside **1 **bearing a syringyl moiety, in addition to four phenylethanoid glycosides **2−5**, two benzoxazinoid glucosides **6, 7**, and two aliphatic alcohol glycosides **8, 9**.

## 2. Results and Discussion

**Figure 1:**
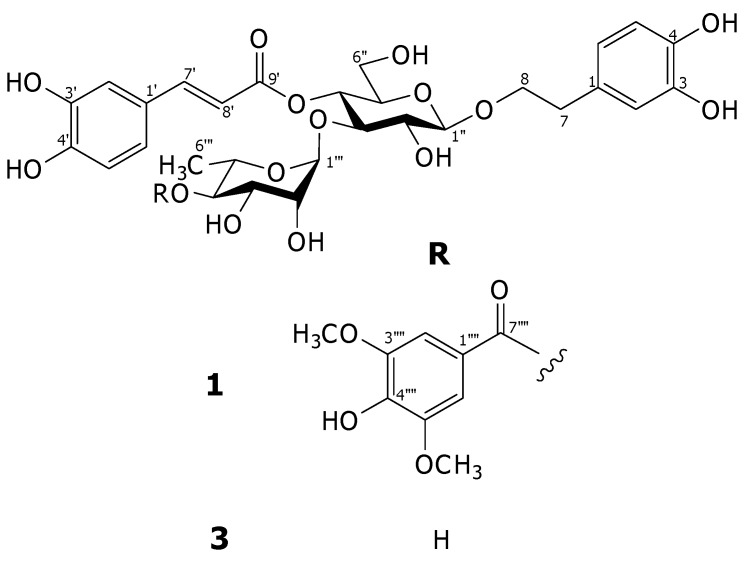
Structures of compounds **1** and **3**.

The methanolic extract of the aerial portions of *A. montanus* was suspended in H_2_O and partitioned with Et_2_O. The aqueous layer was applied to a Diaion HP-20 column, and eluted successively with H_2_O, MeOH and Me_2_CO. The fraction eluted with MeOH was separated by a combination of chromatographic techniques to afford a new phenylethanoid glycoside **1** (Figure 1) together with eight known compounds, identified as decaffeoylverbascoside (**2**), verbascoside (**3**), isoverbascoside (**4**), leucosceptoside A (**5**), (2*R*)-2-*O*-β-D-glucopyranosyl-2*H*-1,4-benzoxazin-3(4*H*)-one (HBOA-Glc, **6**), (2*R*)-2-*O*-β-D-glucopyranosyl-4-hydroxy-2*H*-1,4-benzoxazin-3(4*H*)-one (DIBOA-Glc, **7**), (3*R*)-1-octen-3-ol-3-*O*-β-D-xylopyranosyl-(1→6)-*O*-β-D-glucopyranoside (**8**) and ebractatoside B (**9**) by comparison of physical data with literature values and spectroscopic evidence [2,4,12].

**Figure 2:**
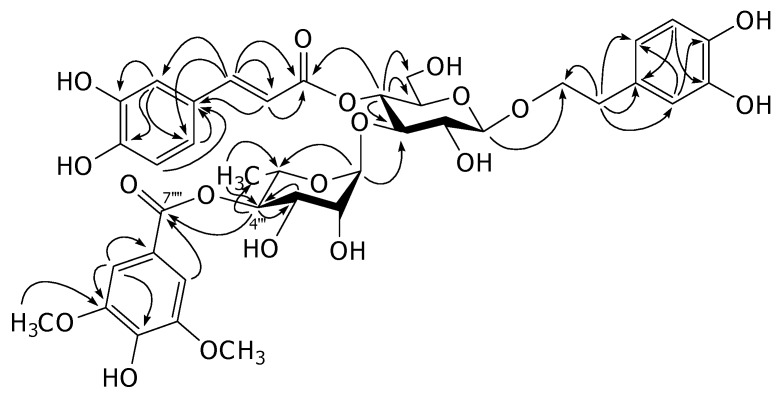
The HMBC correlations of acanmontanoside (**1**).

Compound **1** was isolated as an amorphous powder. Its molecular formula was determined to be C_38_H_44_O_19_ by high resolution electrospray ionization (HR-ESI) mass spectrometric analyses. Inspection of the ^1^H-NMR spectrum indicated that this compound is a verbascoside derivative from the chemical shifts of two sets of ABX aromatic ring systems at δ_H_ 6.57 (*br d*, *J* = 7.2 Hz), 6.69 (*d*, *J* = 7.2 Hz) and 6.71 (*br s*) for 3,4-dihydroxy-β-phenylethoxyl moiety, and at δ_H_ 6.43 (*br d*, *J* = 8.3 Hz), 6.59 (*d*, *J* = 8.3 Hz) and 6.86 (*br s*) for caffeoyl moiety, two *trans*-olefinic protons at δ_H_ 6.21 and 7.52 (each *d*, *J* = 16.0 Hz), two anomeric protons at δ_H_ 4.39 (*d*, *J* = 7.9 Hz) for β-glucose and δ_H_ 5.37 (*br s*) for α-rhamnose. In addition, the signals of 1,3,4,5-tetrasubstituted symmetrical aromatic ring at δ_H_ 7.22 (2H, *s*) and two equivalent methoxy protons at δ_H_ 3.86 (6H,*s*) for an additional group were observed. This functional group was assigned to be a syringyl moiety from the ^13^C-NMR signals at δ_C_ 108.5 (2C), 121.5, 142.1 and 148.8 (2C) for the aromatic ring, two methoxy carbons at δ_C_ 57.0 and one ester carbonyl carbon at δ_C_ 168.0 [1]. Comparison of the ^13^C-NMR chemical shifts of this compound with those of verbascoside (**3**) revealed the downfield shift of C-4"' (+2.5 ppm) together with the upfield shifts of C-3"' (−1.3 ppm) and C-5"' (−2.4 ppm) of the rhanmopyranosyl unit indicating that the syringyl group is an ester located at C-4"' of the rhamnopyranosyl moiety [4]. The assignments were supported by the results from COSY, HMQC and HMBC experiments. In the HMBC spectrum, the significant correlation was found between H-4"' and C-7"" as illustrated in Figure 2. Therefore, the structure of this compound was elucidated as 4"'-*O*-syringyl-verbascoside, namely acanmontanoside.

## 3. Experimental

### 3.1. General

NMR spectra were recorded in CD_3_OD using a JEOL JNM α-400 spectrometer (400 MHz for ^1^H-NMR and 100 MHz for ^13^C-NMR). MS values were obtained on a Bruker Micro TOF-LC mass spectrometer. Optical rotations were measured with a JASCO P-1020 polarimeter. For column chromatography, silica gel 60 (70–230 mesh, no. GE0049, Scharlau Chemie S.A.), RP-18 (50 μm, YMC), and Diaion HP-20 (Mitsubishi Chemical Industries Co. Ltd.) were used. Preparative HPLC was carried out on an ODS column (250 × 20 mm i.d., Nacalai Tesque, Inc.) with a Jasco RI-2031 refractive index detector. The flow rate was 6 ml/min. The solvent systems were: I) EtOAc; II) EtOAc-MeOH (9:1); III) EtOAc-MeOH-H_2_O (40:10:1); IV) EtOAc-MeOH-H_2_O (70:30:3); V) 10−50% aqueous MeOH; VI) 7% aqueous MeCN; VII) 10% aqueous MeCN; VIII) 20% aqueous MeCN; and 25% aqueous MeCN. The spraying reagent used for TLC was 10% H_2_SO_4_ in 50% EtOH.

### 3.2. Plant Material

The aerial part of *A. montanus* (Nees) T. Anderson was collected from the Botanical Garden of the Faculty of Pharmaceutical sciences, Khon Kaen University in December 2005. The plant was identified by Mr. Bamrung Tavinchiua of the Department of Pharmaceutical Botany and Pharmacognosy, Faculty of Pharmaceutical Sciences, Khon Kaen University, Thailand. A voucher specimen (TK-PSKKU-0053) is deposited in the Herbarium of the Faculty of Pharmaceutical Sciences, Khon Kaen University, Thailand.

### 3.3. Extraction and Isolation

Dried aerial portions of *A. montanus* (1.0 kg) were extracted with MeOH three times (5.0 L each) at room temperature. After removal of the solvent by evaporation, the greenish residue (105.1 g) was suspended in H_2_O and partitioned with Et_2_O three times (1.0 L each). The aqueous layer was applied to a column of Diaion HP-20 and eluted successively with H_2_O, MeOH, and Me_2_CO. The fraction eluted with MeOH (10.5 g) was concentrated to dryness and subjected to a silica gel column using solvent systems I (3.0 lit), II (5.0 lit), III (3.0 lit), and IV (1.0 lit). Six fractions were collected (A to F). Fraction A (2.1 g) was applied to a column of RP-18 using solvent system V to provide six fractions. Fractions A-4 was purified by preparative HPLC-ODS with solvent system VIII to yield verbascoside (**3**, 34.3 mg), isoverbascoside (**4**, 36.6 mg). Fraction A-4 was purified by preparative HPLC-ODS with solvent system VIII to give leucosceptoside A (**5**, 32.3 mg), and the new phenylethanoid compound, acanmontanoside (**1**, 34.5 mg). Fraction B (1.4 g) was subjected to a column of RP-18 using solvent system V to afford five fractions. Fraction B-2 was purified by preparative HPLC-ODS with solvent system VII to provide (2*R*)-2-*O*-β-D-glucopyranosyl-2*H*-1,4-benzoxazin-3(4*H*)-one (**6**, 15.6 mg). Fraction C (1.4 g) was separated on a column of RP-18 using solvent system V to give nine fractions. Fraction C-1 was further purified by preparative HPLC-ODS with solvent system VI to provide decaffeoylverbascoside (**2**, 55.5 mg). Fraction C-2 was purified by preparative HPLC-ODS with solvent system VII to obtain (2*R*)-2-*O*-β-D-glucopyranosyl-4-hydroxy-2*H*-1,4-benzoxazin-3(4*H*)-one (**7**, 172.0 mg). Fraction C-7 was further purified by preparative HPLC-ODS with solvent system IX to afford (3*R*)-1-octen-3-ol-3-*O*-β-D-xylopyranosyl-(1→6)-*O*-β-D-glucopyranoside (**8**, 37.0 mg). Fraction D (2.2 g) was similarly separated on a column of RP-18 using solvent system V to afford eight fractions. Fraction D-6 was purified by preparative HPLC-ODS with solvent system IX to provide ebracteatoside B (**9**, 32.2 mg).

### 3.3. Acanmontanoside *(**1**)*

Amorphous powder, 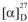 −157.5 (MeOH, *c* = 0.70); ^1^H- and ^13^C-NMR (CD_3_OD): see Table 1; HRESIMS, *m/z*: 803.2408 [M-H]^− ^(calcd for C_38_H_43_O_19_:803.2404).

**Table 1:** NMR Spectroscopic data of acanmontanoside (**1**, CD_3_OD).

No.	^13^C			^1^H
	3	1		1
*Aglycone*				
1	131.5	131.6		
2	117.1	117.2		6.71 (1H, *br s*)
3	146.1	146.2		
4	144.6	144.7		
5	116.3	116.4		6.69 (1H, *d*, *J* = 7.2 Hz)
6	121.3	121.4		6.57 (1H, *br d*, *J* = 7.2 Hz)
7	36.5	36.6		2.80 (2H, *t*, *J* = 7.3 Hz)
8	72.2	72.2		3.74 (1H)^a^
				4.05 (1H)^a^
*Caffeoyl moiety*				
1'	127.7	127.3		
2'	115.2	114.9		6.86 (1H, *br s*)
3'	149.7	149.8		
4'	146.8	146.7		
5'	116.5	116.4		6.59 (1H, *d*, *J* = 8.3 Hz)
6'	123.2	123.2		6.43 (1H, *br d*, *J* = 8.3 Hz)
7'	148.0	148.1		7.52 (1H, *d*, *J* = 16.0 Hz)
8'	114.7	114.5		6.21 (1H, *d*, *J* = 16.0 Hz)
9'	168.3	168.3		
*Glc*				
1''	104.2	104.2		4.39 (1H, *d*, *J* = 7.9 Hz)
2''	76.2	76.5		3.45 (1H, *dd*, *J* = 7.9, 8.4 Hz)
3''	81.6	80.7		3.92 (1H, *dd*, *J* = 8.4, 9.3 Hz)
4''	70.4	70.2		4.99 (1H, *dd*, *J* = 9.3, 9.4 Hz)
5''	76.0	76.0		3.56 (1H, *m*)
6''	62.4	62.4		3.54 (1H)^a^
				3.64 (1H)^a^
*Rha*				
1'''	103.0	102.3		5.37 (1H, *br s*)
2'''	72.3	72.3		3.72 (1H)^a^
3'''	72.0	70.7		3.94 (1H)^a^
4'''	73.8	76.3		5.02 (1H, *dd*, *J* = 9.7, 9.7 Hz)
5'''	70.6	68.2		3.86 (1H)^a^
6'''	18.4	18.7		1.05 (3H, *d*, *J* = 6.2 Hz)
*Syringyl moiety*				
1''''		121.5		
2'''', 6''''		108.5		7.22 (2H, *s*)
3'''', 5''''		148.8		
4''''		142.1		
7''''		168.0		
MeO-3'''', 5''''		57.0		3.86 (6H, *s*)

^a^ Chemical shifts obtained approximately by HMQC.

## 4. Conclusions

The present study isolated phenylethanoid glycosides **1-5**, benzoxazinoid glucosides **6, 7**, and aliphatic alcohol glycosides **8, 9** from the aerial parts of *A. montanus*. These types of compounds have previously been reported in *Acanthus* species [1,2,3,4,5]. It provides further confirmation of the typical profile of secondary metabolites found in this genus. 
